# Oral Health in Patients with Inflammatory Bowel Diseases Qualified for Biologic Treatment

**DOI:** 10.3390/ijerph192315584

**Published:** 2022-11-24

**Authors:** Kacper Nijakowski

**Affiliations:** Department of Conservative Dentistry and Endodontics, Poznan University of Medical Sciences, 60-812 Poznan, Poland; kacpernijakowski@ump.edu.pl

## 1. Introduction

Oral health is closely linked to general health status in the form of a bidirectional relationship. Poor oral health, caused by inadequate dental hygiene and care, can aggravate the course of systemic diseases [1,2,3]. Similarly, systemic diseases or accompanying therapies may be reflected in the form of oral manifestations [4,5]. It is essential to emphasise the importance of dental prevention and care for all age groups, from children to the elderly [6,7,8].

Occasionally, it is the dentist who first notices the symptoms of a systematic disease. Patients taking a variety of medicines may experience xerostomia, which is a common side effect of oral conditions [9]. Therefore, regular dental examinations with oral hygiene instructions are indispensable today as a preventive measure [10,11]. In addition, the outbreak of the COVID-19 pandemic has had a significant impact on dental treatment, with the first wave requiring radical surgical interventions in patients suffering from toothache [12,13]. This event reminded us that preventing diseases, including those affecting the oral cavity, should always be a priority.

Consequently, this Special Issue will be focused on an interdisciplinary approach to oral health and disease prevention.

## 2. Oral Health Status in Patients with Inflammatory Bowel Diseases Qualified for Biologic Treatment

Previous studies investigating oral health status in patients with inflammatory bowel diseases (IBD) have shown heterogeneous results. Based on the conducted meta-analysis [14], my colleagues and I found that both Crohn’s disease (CD) and ulcerative colitis (UC) patients demonstrated an increased odds for the coincidence of periodontal disease compared with healthy subjects, of more than 2- and 3-fold, respectively. Only some studies reported a significantly higher prevalence of periodontitis observed in adult IBD patients [15,16,17,18]. Among the important predisposing factors for periodontal disease, the authors mentioned active forms of systemic disease (e.g., CD with associated perianal lesions) or smoking.

Similarly, children and adolescents with IBD presented an increased incidence of periodontal disease and dental caries, despite oral hygiene indices comparable to those of the healthy subjects [19]. In contrast, other researchers observed higher values of plaque indices in IBD patients but no significant differences in caries and periodontal status between the study and control groups [20]. However, in the most recent studies from 2022, the authors found that the progression of periodontitis is closely associated with a worsening of the course of IBD [21,22]. Therefore, preventive and therapeutic strategies concerning the oral–gut axis should be imposed in IBD patients.

My current research projects have focused on changes in oral health status and the salivary parameters of oral immunity in IBD patients, especially those qualified for biologic treatment [23]. In this group, the incidence of caries was not high—the median number of active carious lesions was only 1. In addition, the caries treatment index had high values, regardless of the IBD form (0.86 [0.69–1.00]). Significantly more teeth with fillings were observed in UC patients (8.5 [5.0–11.5] vs. 5.0 [3.0–8.0], *p*-value = 0.027). Most of the studied patients had complete dental arches, with at most single missing teeth.

Importantly, the study group represented good oral hygiene status, and no significant differences were observed between CD and UC patients. Lower values of indices indicating better oral hygiene status were found in CD patients (for approximal plaque index 23.7 [11.5–37.5] vs. 36.8 [22.3–47.4], *p*-value = 0.145, and for plaque index 0.33 [0.17–0.38] vs. 0.44 [0.23–0.58], *p*-value = 0.189). All patients manifested healthy periodontal status (periodontal probing depth 1.42 [1.15–1.58] mm), with at most mild localised gingivitis. Patients with UC had higher gingival index values, close to statistical significance (0.33 [0.21-0.46] vs. 0.25 [0.17–0.29], *p*-value = 0.054). The obtained values of oral hygiene status indices correlated very strongly with periodontal status indices, especially with GI (for API and PlI - R_s_ = 0.837 and 0.931, respectively). A very strong correlation was also found between both determined indicators of oral hygiene (R_s_ = 0.853).

Oral lesions were observed more frequently in the patients with UC (41.67 vs. 23.08%, *p*-value = 0.227). Among the evaluated lesions, mainly those of non-specific nature were noted, such as aphthous erosions and lip angle inflammation. No specific lesions on the oral mucosa were found in patients with CD. A small percentage of patients complained of dry mouth (6.0%).

Moreover, Ślebioda et al. [24] found no significant differences in the level of oral hygiene as well as dental and periodontal status in IBD patients compared with controls. As far as the occurrence of oral mucosal lesions is concerned, recurrent aphthous ulcers were observed with comparable frequency in both IBD forms (27.1% of patients with CD and 32.0% of patients with UC), but much more frequently than in healthy subjects (8.6%). Comparing the patients described in the above report from 2011 with my study group, an improved dental and periodontal status in patients with IBD treated in the Poznan centre can be seen.

## 3. Conclusions

Patients with IBD that qualified for biologic treatment were characterised by good dental and periodontal status as well as a good level of oral hygiene associated with the declared correct habits. On the other hand, the conducted systematic review suggests increased odds of periodontal disease in IBD patients, especially those with UC. Therefore, the relationship between oral health and IBD cannot be clearly defined due to individual, sociodemographic, and environmental cofactors.
